# Landscape of Participant-Centric Initiatives for Medical Research in the United States, the United Kingdom, and Japan: Scoping Review

**DOI:** 10.2196/16441

**Published:** 2020-08-04

**Authors:** Nao Hamakawa, Rumiko Nakano, Atsushi Kogetsu, Victoria Coathup, Jane Kaye, Beverley Anne Yamamoto, Kazuto Kato

**Affiliations:** 1 Department of Biomedical Ethics and Public Policy Graduate School of Medicine Osaka University Suita, Osaka Japan; 2 Graduate School of Human Sciences Osaka University Suita, Osaka Japan; 3 Centre for Health, Law and Emerging Technologies Faculty of Law University of Oxford Oxford United Kingdom; 4 Centre for Health, Law and Emerging Technologies Melbourne Law School University of Melbourne Melbourne Australia

**Keywords:** participant-centric initiatives, patient involvement, patient engagement, participatory research, participatory medicine, information and communication technology, patient participation

## Abstract

**Background:**

Information and communication technology (ICT) has made remarkable progress in recent years and is being increasingly applied to medical research. This technology has the potential to facilitate the active involvement of research participants. Digital platforms that enable participants to be involved in the research process are called participant-centric initiatives (PCIs). Several PCIs have been reported in the literature, but no scoping reviews have been carried out. Moreover, detailed methods and features to aid in developing a clear definition of PCIs have not been sufficiently elucidated to date.

**Objective:**

The objective of this scoping review is to describe the recent trends in, and features of, PCIs across the United States, the United Kingdom, and Japan.

**Methods:**

We applied a methodology suggested by Levac et al to conduct this scoping review. We searched electronic databases—MEDLINE (Medical Literature Analysis and Retrieval System Online), Embase (Excerpta Medica Database), CINAHL (Cumulative Index of Nursing and Allied Health Literature), PsycINFO, and Ichushi-Web—and sources of grey literature, as well as internet search engines—Google and Bing. We hand-searched through key journals and reference lists of the relevant articles. Medical research using ICT was eligible for inclusion if there was a description of the active involvement of the participants.

**Results:**

Ultimately, 21 PCIs were identified that have implemented practical methods and modes of various communication activities, such as patient forums and use of social media, in the field of medical research. Various methods of decision making that enable participants to become involved in setting the agenda were also evident.

**Conclusions:**

This scoping review is the first study to analyze the detailed features of PCIs and how they are being implemented. By clarifying the modes and methods of various forms of communication and decision making with patients, this review contributes to a better understanding of patient-centric involvement, which can be facilitated by PCIs.

**International Registered Report Identifier (IRRID):**

RR2-10.2196/resprot.7407

## Introduction

The use of information and communication technology (ICT) is increasing in all aspects of health care delivery and medical research, enabling vast amounts of data to be accumulated and analyzed at an unprecedented rate. Furthermore, a growing number of people are participating in research using smart devices, such as smartphones, tablets, and wearable devices [1]. Participation in medical research using ICT is expected to increase in the future. At the same time, patient-reported outcomes, where the patient reports directly on his or her condition, such as pain, fatigue, and quality of life, are recognized as important for both clinical and research settings [2,3]. This new trend in medicine also facilitates the use of ICT in the medical research fields.

In parallel, attitudes toward medical research are also shifting to more active involvement of research participants. This emerging model of research, in which researchers and participants collaborate through the research process, is gaining momentum internationally. Several research funders, such as INVOLVE in the United Kingdom, which was established by the National Institute for Health Research (NIHR) to support public involvement in the National Health Service (NHS), and the Patient-Centered Outcomes Research Institute (PCORI) in the United States, are known as leading organizations promoting the involvement of research participants [4].

The definition of patient and public involvement and engagement has not yet been established. There is no designated terminology to describe the active involvement of participants in research. According to INVOLVE, *involvement* is defined as “research that is being carried out ‘with’ or ‘by’ members of the public, rather than ‘to,’ ‘about,’ or ‘for’ them,” while *engagement* indicates “information and knowledge about research is provided and disseminated” [5]. On the other hand, PCORI in the United States uses the term *engagement* in the following way: “The meaningful involvement of patients, caregivers, clinicians, and other healthcare stakeholders throughout the entire research process” [6]. Another example of the fluidity of the terminology can be seen in a consensus statement on patient and public involvement with data-intensive health research, which was developed by an international group of experts; they use the term *public involvement and engagement* [7]. While the terminology may change, a shared sentiment in all these documents is that they define patients or the public as experts in their personal knowledge and experiences. Collaborating with patients and the public is expected to improve research quality and relevance, ensure transparency and accountability, and foster innovation and research [5-9].

In this review, we use the definition from INVOLVE [5], where *involve* or *involvement* refers to a status where participants play an active role in medical research, beyond the level of simply being a participant to take part in research or inputting their data [10]. For instance, we hoped to find examples where participants collaborate with researchers and have influence over research design, analysis, management, and/or dissemination. While recognizing the diversity of approaches to involvement, here, we are not including activities such as the raising of awareness of research by patients or the creation of events, such as workshops and festivals to engage with the public [11].

Participant-centric initiatives (PCIs) are new initiatives that employ ICT for facilitating active involvement in research and are defined as “digital tools, platforms, or projects that have been developed to help participants become more actively involved in the research process” [12]. PCIs have the potential to provide a number of benefits to both participants and researchers, including facilitating participant recruitment and retention, providing the basis for long-term partnerships, and sustaining public confidence in research [13]. In addition to this, the interactive interface facilitates communication between research participants and researchers throughout the research process and allows participants to be placed at the center of the decision-making process [13]. Furthermore, it is believed that PCIs can address issues toward protecting individual interests by mediating participants’ control and choice within diverse research contexts [12].

While examples and features of PCIs have been reported by Anderson et al [12] and Kaye et al [13], in 2012, the definition of PCIs and methods of involvement that are promoted through PCIs had not yet been established. Moreover, the status and characteristics of PCIs since they were first outlined in these papers have not been reported. We believed that it was important to capture the current landscape of PCIs and describe detailed characteristics and methods of participant involvement.

By applying a scoping review methodology, we examined PCIs that have been implemented in the United States, the United Kingdom, and Japan, and we systematically analyze their detailed functions and features. The reason for selecting these countries for the study was that the United States and the United Kingdom have been actively advancing patient-centric approaches in medical research, and the majority of reported PCIs in previous research was located in these two countries [12,13]. Our aim was to build on the existing research by updating the PCI landscape for the United Kingdom and the United States and by further contributing to the literature by adding Japan. Japan is a leading country in health research and practice, and we were well positioned to access the literature. While a traditional model of medical research remains pervasive in Japan [14], there is a clear shift toward exploring or prioritizing patient and public involvement, as seen in recent statements made by the Japan Agency for Medical Research and Development, a major medical funding agency [15]. Given this new focus, it was timely to search and explore the current landscape of PCIs available in Japan. We felt that focusing our attention on these three countries would make an important contribution to the literature on PCIs.

Therefore, the aims of this study were (1) to identify existing PCIs used for medical research in the United States, the United Kingdom, and Japan, (2) to describe recent trends and features of PCIs, and (3) to highlight the methods of participant involvement facilitated by PCIs.

## Methods

### Overview

The methodology followed here is based on the previously published study protocol [16]. The scoping review was considered the most appropriate method to address the aims of the study for the following reasons. Firstly, the scoping review is recommended, as it is particularly relevant to disciplines with emerging evidence [17]. Unlike a systematic review, we are not trying to answer a specific question, but rather “examine the extent, range, and nature” of PCIs [18]. Secondly, a scoping review provides comprehensive search methods to incorporate a range of study designs in both published and grey literature. The scoping review methodological framework described by Levac et al [17] was applied to this study.

### Identifying the Research Question

According to the features of PCIs described by Anderson et al [12], it is not necessary to designate the field of research upfront, but we decided to limit our study to PCIs generating data for medical research. In addition, citizen science, which we define here as a research activity directed and conducted by citizens without collaboration with researchers, was considered beyond our remit [19]; therefore, we focused on interaction between participants and researchers using PCIs.

In this study, *medical research* refers to any research involving human subjects aimed at improving clinical outcomes, including prevention; understanding the etiology of diseases and/or effect of treatments; and improving the quality of life of patients.

### Identifying Relevant Studies

We first conducted a literature search in June 2017 in the following databases: MEDLINE (Medical Literature Analysis and Retrieval System Online), Embase (Excerpta Medica Database), CINAHL (Cumulative Index of Nursing and Allied Health Literature), PsycINFO, and Ichushi-Web. We conducted our search by using subject headings and keywords. Search terms included a combination of keywords and subject headings such as Medical Subject Headings (MeSH). Keywords were comprised of the following terms: “participant” AND “centric” OR “centered” OR “centred” OR “engage” OR “involve” OR “collaborat” OR “partner” OR “led” OR “driven” OR “initiat” OR “oriented.” Subject headings for “participation,” “technology,” and “research” were searched and adjusted to best meet the requirements of each database. The detailed search strategies and history are shown in Multimedia Appendix 1. A subject librarian was consulted and provided guidance on the search strategy. We also conducted a cited literature search using the Web of Science and by hand-searching of key journals—*Digital Health* and *The Journal of mHealth*—in August 2017.

A grey literature search was also conducted using Open Grey in December 2017, and a website search was conducted using Google and Bing from April to June 2018. The grey literature and website searches were conducted by using the same search keywords as in the literature database search. Some searches showed a large number of items, for example, more than 100,000 hits. However, as it was practically challenging to identify relevant websites by screening all items, we took a pragmatic approach and screened websites that had appeared within the first 50 results for each keyword.

### Study Selection

The relevant articles and websites were screened based on the inclusion criteria described in Textbox 1. These criteria were formulated based on the description of the PCIs [12,13], and some criteria were added by the research team in the process of screening. The articles obtained by the database search were screened by two independent reviewers (NH and RN), and PCIs were identified from this process. The items identified from the grey literature and website searches were screened by NH, and the selection was confirmed by the research team after several rounds of discussion on the screening results.

**Selection criteria for relevant articles and websites.**

Inclusion criteria:

- Research enables participants to become actively involved in the research design
- Complies with participant-centric initiative (PCI) features described by Anderson et al [12]: (1) digital device or tool, computer program, or digital platform, and (2) projects that empower participants to engage in the research process
- Articles, documents, or websites published in English or Japanese
- Adult population (ie, over 18 years of age)
- Focuses on medical research purposes
- Available to participants in the United States, the United Kingdom, or Japan

Exclusion criteria:

- Platforms that enable patients to connect and communicate with other patients only
- Platforms that use data for research, but there is no interaction between participants and researchers
- Research activity directed and conducted by citizens without the support of scientists
- Research intended to improve the efficiency of clinical practices or to develop tools for health care services
- Medical research that aims to engage with participants without using a digital platform

### Data Extraction

The features of PCIs were extracted and mapped from the relevant articles and information on the websites of each PCI. The data extraction was completed by NH, and the preliminary results were reviewed by research study members to ensure validity. Characteristics to extract included the following: characteristics of PCI websites, type of medical research, and method of involvement. The list of data elements that were extracted are shown in Multimedia Appendix 2.

### Collating, Summarizing, and Reporting the Results

The PRISMA (Preferred Reporting Items for Systematic Reviews and Meta-Analyses) flowchart template [20] was applied to report on the search process. The key characteristics of the included PCIs were summarized in results tables and charts. The key findings were described in a summary report before disseminating to expert panel members.

### Consultation With Expert Panel

A consultation is recommended as an optional stage in conducting a scoping review [17]. We carried out a consultation in January 2019 with a small number of experts to receive feedback about the obtained results, including additional information and perspectives. Using our research network, the expert panel included four researchers and a patient representative with expertise in patient and public involvement, health care and digital technologies, and clinical cohort studies from the United States, the United Kingdom, Australia, and Japan.

## Results

### Identifying Relevant Studies and Study Selection

**Figure 1:**
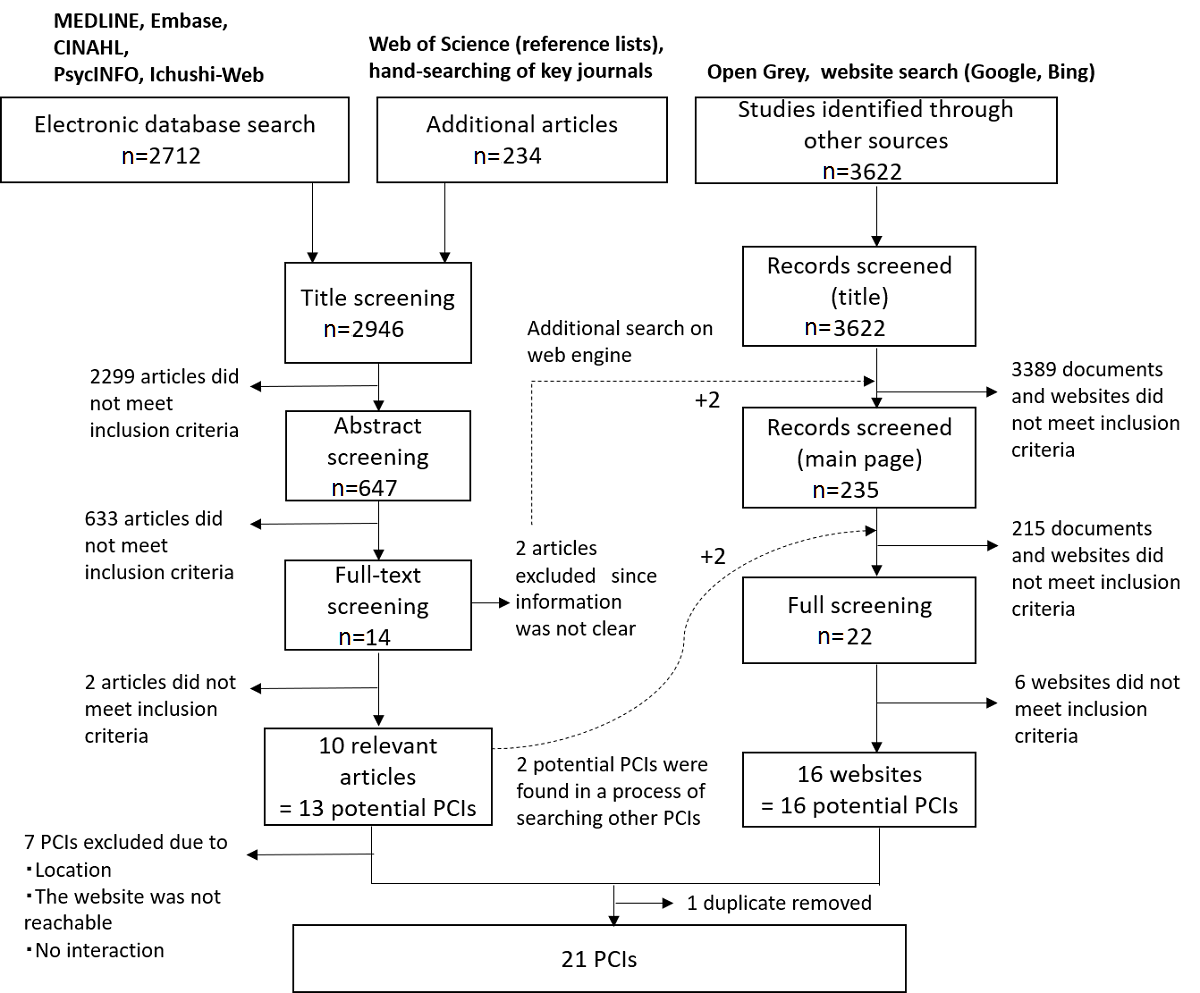
Flowchart for study selection. MEDLINE: Medical Literature Analysis and Retrieval System Online; Embase: Excerpta Medica Database; CINAHL: Cumulative Index of Nursing and Allied Health Literature; PCI: participant-centric initiative.

The search results and screening process are shown in Figure 1. The screening process can be divided into two flowcharts: the database search and the grey literature search.

In the database search, 10 relevant articles [1,12,13,21-27] remained after the title, abstract, and full-text screening, and the descriptions of 13 potential PCIs were found in those papers. Within these 13 potential PCIs, 3 had been implemented in nontargeted countries (ie, Italy, the Netherlands, and Iceland), and 3 websites were not accessible, possibly due to the termination of the projects. Only 1 website focused on releasing personal genetic data openly to the public; we considered this beyond the inclusion criteria of this scoping review because all participants were scientists, and they were not aiming at collaborating with patients or citizens. As a result, 6 PCIs satisfying the inclusion criteria were identified in the database search.

The grey literature and website searches were conducted in an iterative process reflecting the results of the database search that had been performed earlier. In total, 3622 documents and websites were screened, but none described the implementation of PCIs for medical research. However, 16 additional PCIs were identified by a supplementary search of 2 excluded articles [28,29] from the database search. Both articles were related to the PCORI. Another 2 potential PCIs were discovered while searching for information on other PCIs that had been found in the database search. In total, 16 PCIs were identified in the website search. After removing duplicates, 21 PCIs [30-50] were ultimately identified in the obtained results (see Table 1). The detailed characteristics of these 21 PCIs are also shown in Multimedia Appendix 3.

**Table 1:** Participant-centric initiatives (PCIs) identified by our scoping review.

No.	Name of PCI	Location	Type of organization	Areas of focus	Launch year	Number of users^a^
1	23andMe [30]	United States	Industry	Diverse (more than 230)	2006	>1,200,000
2	PatientsLikeMe [31]	United States	Industry	Diverse (more than 2800)	2006	>600,000
3	PEER^b^ [32]	United States	NPO^c^	Diverse (about 50)	2014	>15,000
4	GenomeConnect [33]	United States	Research institute	Diverse (genetic disorders)	2014	1400
5	RUDY^d^ [34]	United Kingdom	University	Fibrous dysplasia, vasculitis, osteogenesis imperfect, etc	2014	993
6	MoodNetwork [35]	United States	Research hospital	Mood disorders	2015	Unknown
7	mPower [36]	United States	NPO	Parkinson disease	2015	15,000
8	J-RARE [37]	Japan	NPO	Distal myopathy, relapsing polychondritis, Marfan syndrome, etc	2013	≥47
9	ABOUT Network [38]	United States	NPO and university	Hereditary breast cancer	2016	10,500
10	Arthritis Power [39]	United States	NPO	Rheumatoid arthritis, fibromyalgia, inflammatory bowel disease (IBD), etc	2014	15,365
11	IBD^e^ Partners [40]	United States	NPO and university	Crohn disease	2011	15,680
12	Rare Epilepsy Network (REN) [41]	United States	NPO	Rare epilepsy	2014	1392
13	COPD PPRN^f^ [42]	United States	NPO	Chronic obstructive pulmonary disease and asthma	2014	75,000
14	Health eHeart [43]	United States	University	Cardiovascular diseases	2013	75,000
15	IAN^g^ [44]	United States	NPO	Developmental disorder	2006	>20,000
16	iConquerMS (multiple sclerosis) [45]	United States	NPO	Multiple sclerosis	2015	≥3100
17	AD-PCPRN^h^ [46]	United States	Research hospital	Alzheimer disease and dementia	2014	57,000
18	NephCure Kidney Network Patient Registry [47]	United States	NPO	Primary nephrotic syndrome	2014	666
19	PI^i^ CONNECT [48]	United States	NPO	Primary immunodeficiency	Unknown	5040
20	V-PPRN^j^ [49]	United States	University	Behçet disease, vasculitis, polyarteritis nodosa, etc	Unknown	Unknown
21	MyApnea [50]	United States	Research hospital	Sleep apnea	2013	12,677

^a^The number of registrants (ie, users) is based on information publicly available in July 2018.

^b^PEER: Promise for Engaging Everyone Responsibly.

^c^NPO: nonprofit organization; includes patient organizations and research organizations.

^d^RUDY: Rare and Undiagnosed Diseases Study.

^e^IBD: Inflammatory Bowel Disease.

^f^COPD PPRN: Chronic Obstructive Pulmonary Disease Patient-Powered Research Network.

^g^IAN: Interactive Autism Network.

^h^AD-PCPRN: Alzheimer's Disease Patient- and Caregiver-Powered Network.

^i^PI: Primary Immunodeficiency.

^j^V-PPRN: Vasculitis Patient-Powered Research Network.

### Difficulties in Searching for Relevant Studies

During the database search, it became clear that there were no designated subject headings to describe features of the PCIs within this emerging field; for instance, the existing MeSH do not have terms to include “participant-centric,” “engagement,” or “involvement”; alternatively, “patient participation” was suggested as the only MeSH option. The subject heading is typically used as an effective search tool in a database search. In this case, however, we had to combine keywords to search for articles relevant to PCIs. The lack of effective search terms may reflect the novelty of the field. For the same reason, the results of grey literature and website searches using Google and Bing were also limited.

Many of the documents that were excluded in the screening process were related to patients’ decision making or participation in clinical practice or medical interventions, including test screening, rather than medical research. Another major category of excluded items concerned the digitization of research and health care with the aim of developing tools for services, with no description of the involvement of participants.

### Results of the Expert Panel

After the consultation, all members of the expert panel commented on the obtained results. Overall, they considered the results “important and well worthy of publication” and the search strategy as “sensible.” In addition, the experts gave feedback on the analysis and discussion sections of the paper. The feedback was incorporated into our analysis of results and the discussion.

There were also suggestions on potential PCIs we had not identified through the literature search. A total of 5 potential platforms were suggested by two members of the panel—one from Japan and one from the United Kingdom. Moreover, one member suggested that disease registries were underrepresented in the obtained results. The main reasons that we did not pick up these registries with our literature search is that any scientific papers generated by them appeared after our cutoff point or they did not indicate active involvement of patients in existing papers.

In response, we screened the registries listed on the National Institutes of Health (NIH) website. A total of 62 registries were screened by NH and AK. As a result, we identified 5 PCIs in the United States and Japan, which had not come out in our original literature search. Moreover, an anonymous reviewer suggested an additional PCI during the peer-review process. As we had decided that the results of the literature searches would be our endpoint for this study, these additional PCIs are offered here as supplementary data (see Multimedia Appendix 4). It is noteworthy, however, that the features of these PCIs do not change the overall landscape we describe as the main endpoint of the study.

### Recent Trends in PCIs

PCIs have been implemented for research focusing on various diseases, including rare diseases, mood disorders, heart diseases, and dementia. Of the 21 kinds of PCIs identified in this scoping review, 4 (19%)—23andMe, PatientsLikeMe, PEER (Promise for Engaging Everyone Responsibly), and GenomeConnect—had features that included a variety of disease areas and had cross-cutting registry functions ranging from dozens to thousands of diseases. One of them was a direct-to-consumer genetic testing company (ie, 23andMe). In these PCIs, multiple kinds of medical research were being conducted. Furthermore, these 4 PCIs aimed to match the genomic information of research participants with their disease phenotype. On the contrary, the main registration objectives of the other 17 PCIs for medical research concerned specific disease areas; some of them had gradually expanded their disease areas of focus, such as RUDY (Rare and Undiagnosed Diseases Study) and V-PPRN (Vasculitis Patient-Powered Research Network).

**Figure 2:**
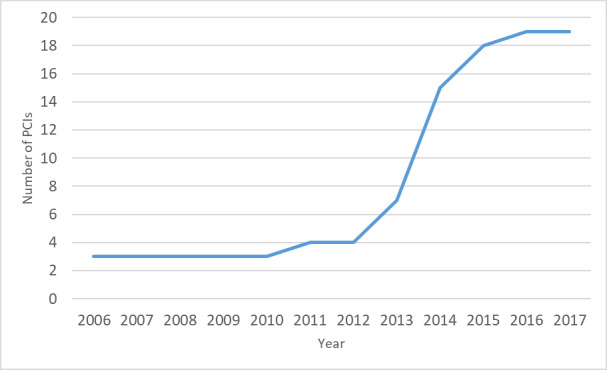
The trend in the number of participant-centric initiatives (PCIs) identified in the scoping review, cumulative total by year. NB: For 2 PCIs, the launch year was unknown.

Examining the year in which each PCI was established, our results show a dramatic upward trend from 2013 onwards (see Figure 2).

### Medical Research Facilitated by PCIs

The medical research conducted using digital tools, such as personal computers and smartphones, had one or both of the following purposes:

1. Understanding the symptoms: changes in the presentation of symptoms over time, genetic information and disease phenotype matching, daily changes of conditions, and health conditions associated with everyday life, including exercise and diet.
2. Comparison of treatment effectiveness: a case-control study comparing the effects of interventions, such as comparing medication records with symptoms or the impact of an online exercise class.

We found that at least 277 articles had already been published by several PCI research groups by January 2018, including some in major scientific journals [33-36,40,50-52]. Among them, PatientsLikeMe and 23andMe are promoting research covering various disease areas and have published more than 100 papers [51,52]. All PCIs were used to facilitate participation in substudies or provide opportunities to join upcoming research.

Table 2 shows the specific data collected by each PCI. Overall, the majority of PCIs had analyzed questionnaires on symptoms and quality of life. Functions to upload the results of DNA testing and electronic health record data were also implemented in 7 PCIs, corresponding to one-third of the total. Other functions included tracking personal data, such as body temperature, blood pressure, and weight. A total of 3 PCIs had an interface to collect data using new functions on smartphones and wearable devices, such as those relating to movement and voice.

**Table 2:** Types of data collected by participant-centric initiatives (PCIs).

Types of data	Number of PCIs (N=21), n (%)
DNA test result	7 (33)
Closed-ended questionnaire	17 (81)
Open-ended questionnaire	5 (24)
Treatment and medication	16 (76)
Motion and voice	3 (14)
Self-reported measurement	4 (19)
Electronic health record	5 (24)^a^

^a^In addition to the 5 PCIs that collected electronic health record data, 2 were in preparation.

### Model of Informed Consent

Table 3 shows the result of consent models that were indicated on each of the websites. A *specific consent model*—a participant’s consent is requested each time they participate in a new study—was used for approximately half the PCIs (12/21, 57%). On the contrary, a *broad consent model* was used in 1 PCI (5%) that was developed by a for-profit organization (ie, PatientsLikeMe).

In total, 3 PCIs out of 21 (14%)—PEER, RUDY, and J-RARE—implemented a *dynamic consent model*. Dynamic consent is a new model that enables participants to change their consent status over time online [53]. Furthermore, participants can also select the range of data sharing and methods of communication. There were 5 PCIs out of 21 (24%) where the consent process was unclear.

**Table 3:** Model of consent implemented in participant-centric initiatives (PCIs).

Types of data	Number of PCIs (N=21), n (%)
Specific consent	12 (57)
Broad consent	1 (5)
Dynamic consent	3 (14)
Treatment and medication	16 (76)
Unknown	3 (14)

### Various Communication Activities

By examining the activities undertaken by the 21 PCIs, it became clear that various modes of communication had been used, including patient forums, webinars, and dialogue (ie, patients and researchers exchanging messages online) (see Table 4). Moreover, a majority of the PCIs had used multiple social media accounts. Some PCIs had disseminated information through blogs and newsletters. Others had their own digital interface that allowed participants to take part in networking with researchers and other participants. Out of 21 PCIs, 2 (10%) had been conducting face-to-face forums or seminars.

**Table 4:** Modes and methods of communication for the participant-centric initiatives (PCIs).

No.	Name of PCI	Patient forum^a^	Webinar^b^	Dialogue^c^	Use of social media for communication				Other modes of communication
					Facebook	Twitter	YouTube	Other	
1	23andMe		✓		✓	✓	✓	✓	News
2	PatientsLikeMe	✓		✓	✓	✓	✓	✓	News
3	PEER^d^	✓			✓	✓	✓		MOSAIC^e^
4	GenomeConnect			Unclear	✓	✓	✓	✓	Newsletter and mailing list
5	RUDY^f^	✓			✓	✓			N/A^g^
6	MoodNetwork	✓		✓	✓		✓		N/A
7	mPower		✓				✓	✓	Patient satisfaction questionnaire
8	J-RARE								Questionnaire
9	ABOUT Network		✓		✓	✓			GAP360^h^
10	Arthritis Power			✓	✓	✓	✓		N/A
11	IBD^i^ Partners		✓	✓	✓	✓	✓	✓	Blog and dashboard for research ideas
12	Rare Epilepsy Network (REN)	✓	✓		✓	✓			Dashboard
13	COPD PPRN^j^			✓					COPD360°
14	Health eHeart			✓	✓	✓		✓	Health eHeart community
15	IAN^k^		✓		✓	✓	✓		N/A
16	iConquerMS (multiple sclerosis)	✓		✓	✓	✓	✓	✓	Newsletter and iConquerMS community
17	AD-PCPRN^l^				✓	✓	✓		
18	NephCure Kidney Network Patient Registry		✓		✓				Patient story and regional volunteer community
19	PI^m^ CONNECT	✓	✓		✓	✓	✓		N/A
20	V-PPRN^n^		✓		✓	✓		✓	N/A
21	MyApnea	✓		✓	✓	✓			Online bulletin board, blog, and personalized report

^a^Included community day, leadership summit, and research forum.

^b^Included content for general use.

^c^Included the sharing of experiences, thoughts, and information with researchers and other patients; networking.

^d^PEER: Promise for Engaging Everyone Responsibly.

^e^MOSAIC: Model of Observational Screening for the Analysis of Interaction and Communication.

^f^RUDY: Rare and Undiagnosed Diseases Study.

^g^N/A: not applicable.

^h^GAP: Generate, Assess, Prioritize, Plan, Perform, and Publish.

^i^IBD: Inflammatory Bowel Disease.

^j^COPD PPRN: Chronic Obstructive Pulmonary Disease Patient-Powered Research Network.

^k^IAN: Interactive Autism Network.

^l^AD-PCPRN: Alzheimer's Disease Patient- and Caregiver-Powered Network.

^m^PI: Primary Immunodeficiency.

^n^V-PPRN: Vasculitis Patient-Powered Research Network.

### Participant Decision Making in Various Research Processes

It became clear that there were various phases in decision making by research participants. First, the 3 PCIs that implemented the dynamic consent model allowed each participant to control the range of data sharing. Most PCIs used an interface that enabled inputs of participant feedback on research and operations or agenda setting by allowing participants to propose new research questions. Furthermore, more than half the PCIs (14/21, 67%) had a governance structure that included participant representatives in the decision-making process of the research design and conduct of the research (see Table 5).

**Table 5:** Decision-making process implemented in participant-centric initiatives (PCIs).

No.	Name of PCI	Data-sharing control	Individual feedback and suggesting research questions^a^	Research design and governance^b^
1	23andMe		✓	
2	PatientsLikeMe		✓	✓
3	PEER^c^	✓	✓	✓
4	GenomeConnect		✓	✓
5	RUDY^d^	✓	✓	✓
6	MoodNetwork		✓	✓
7	mPower		✓	
8	J-RARE	✓		✓
9	ABOUT Network		✓	✓
10	Arthritis Power		✓	✓
11	IBD^e^ Partners		✓	
12	Rare Epilepsy Network (REN)		✓	
13	COPD PPRN^f^			✓
14	Health eHeart		✓	✓
15	IAN^g^		✓	
16	iConquerMS (multiple sclerosis)		✓	✓
17	AD-PCPRN^h^			✓
18	NephCure Kidney Network Patient Registry		✓	✓
19	PI^i^ CONNECT		✓	
20	V-PPRN^j^		✓	
21	MyApnea		✓	✓

^a^Individual comments, suggestions of research questions, decisions of priority, etc.

^b^The main purpose is to determine the overall policy as representative of research participants, such as an advisory board, a steering committee, and a governor group.

^c^PEER: Promise for Engaging Everyone Responsibly.

^d^RUDY: Rare and Undiagnosed Diseases Study.

^e^IBD: Inflammatory Bowel Disease.

^f^COPD PPRN: Chronic Obstructive Pulmonary Disease Patient-Powered Research Network.

^g^IAN: Interactive Autism Network.

^h^AD-PCPRN: Alzheimer's Disease Patient- and Caregiver-Powered Network.

^i^PI: Primary Immunodeficiency.

^j^V-PPRN: Vasculitis Patient-Powered Research Network.

## Discussion

### Principal Findings

In this study, we conducted a scoping review to capture the recent trend and features of PCIs for medical research, in particular, by focusing on active involvement. A total of 21 PCIs were identified by the scoping review. After analyzing the detailed functions and characteristics of each, the landscape of PCIs became clearer. To our knowledge, this is the first scoping review conducted in this emerging area to map the extent and range of PCIs currently available across the United States, the United Kingdom, and Japan.

### Recent Trends in PCIs

The PCIs identified in this study were utilized in medical research in various fields. The number of participants registered for each PCI ranged from approximately 50 to 100,000 (see Table 1). Moreover, it was revealed that there are two types of PCIs in terms of target population and research purpose. A total of 4 PCIs targeted dozens or more diseases, aimed at understanding the relationship between genomic information and clinical phenotype. On the contrary, the other 17 PCIs targeted specific disease areas with the aim of deepening clinical understanding of the symptoms. In addition, PCIs connect participants and researchers with opportunities to join in different medical research studies. Regarding the significant number of participants and the broad targets of research, it is evident that ICT interfaces provide effective ways to promote research without the limitations of conducting research in real time and geographic area.

Another noteworthy trend was the sharp increase in the number of PCIs after 2013. The reasons for the increase may be improvements in technology and greater understanding of the benefits of using digital technologies in health care, as well as an increasing awareness of the importance of engaging with patients. As 12 of the 21 PCIs (57%) were funded by PCORI in the United States, the trend may also have been influenced by the year of its establishment. PCORI was established in 2010 with the aim of funding comparative clinical effectiveness research for patients and those who care for them to make better-informed health decisions [54]. Moreover, PCORI promotes patient engagement to ensure that results are relevant and useful to stakeholders [55,56]. Meanwhile, various factors, such as an increased research focus on patient-reported outcomes [57] and more medical research using smartphones and wearable devices [58], are also thought to be related to this recent trend in PCIs.

There are at least 270 scientific papers published that used data collected by PCIs. Out of 21 PCIs, 2 (10%)—23andMe and PatientsLikeMe—are notable because they have published nearly 100 papers in the area between them; both are organized by for-profit organizations. It is necessary to analyze them further to understand the kinds of research papers that are published by these PCIs and why these for-profit organizations seem to have a greater reach than others.

### Features of PCIs

The features of PCIs have been described in a prior paper [13] as “placing participants in control; using social media technology; promoting active participation; facilitating communication; appealing to public good.” With the results of this scoping review, these features have become clearer. 　

Dynamic consent was implemented in 3 out of 21 PCIs (14%)—PEER, RUDY, and J-RARE—while 12 PCIs (57%) implemented the specific consent model. This result indicates that not all PCIs embedded individual control into the design of the interface.

The use of social media technology, such as information dissemination, was observed in most of the 21 PCIs. This allows participants to see the progress in research and the results. PCIs also generally provide user-friendly platforms that facilitate two-way interaction between participants and researchers and, in some PCIs, between the participants. In more than half of the 21 PCIs, participants were involved in suggesting research questions. These results indicate that PCIs enable participants to play an active role, not only in terms of controlling their own data-sharing settings but also by way of contributing to decision making in research design. They encourage participants to become involved in the agenda setting of the entire research community. A follow-up study is needed to examine the method and evaluation of these involvement activities.

### Definition of PCIs

The definition of PCIs was suggested in prior studies in 2012 [12,13], as described in our introduction. However, while conducting this scoping review, we initially found it difficult to distinguish PCIs from other related digital platforms. By analyzing the obtained results, the essential concept and features of PCIs became clearer. Therefore, in this study, we reconsidered the definition of PCIs with clearer and more rigorous criteria. Our proposed updated definition of PCIs is as follows: (1) research activities use ICT, (2) participants are actively involved in the agenda setting of research, and they play roles beyond those of research subjects or assistants, (3) participants can communicate with researchers interactively throughout the research process, and (4) participants can choose the level of their involvement.

PCIs cultivate an environment to establish collaborative partnerships between participants and researchers. As this *participant-centric* model is still emerging, the number of PCIs that are currently in practice is small, compared with other kinds of medical research that uses ICT. Employing a rigorous criteria of patient involvement, we excluded researcher-led initiatives that aim at collecting data, such as many patient registries and disease registries, because they did not indicate active involvement by patients either on their website or in their published literature. We also excluded patient registries created with the active support of patient organizations if that involvement was limited to recruiting patients to provide data. Moreover, research projects that limit patient involvement to communicating with participants to disseminate information or to respond to inquiries were also excluded, as we did not consider this to be interactive communication. Clarifying exclusions and inclusions as part of the screening process also allowed us to clearly identify the distinctive levels of involvement and the features of PCIs.

### Comparison Between PCIs in the United States, the United Kingdom, and Japan

Of the 21 PCIs identified, 19 (90%) were initiated in the United States. There may be a number of possible reasons for this, some of which suggest avenues for future investigation. Firstly, it is possible that the culture of involving patients and members of the public in research is more established in the United States.

Our results indicated that many of the PCIs were funded by PCORI. The Patient-Powered Research Network is one of the research networks that is operated and governed by patient groups, and it has launched a number of projects using online platforms to collect self-reported data [55,56]. This may suggest that differences in policies of funding organizations have influenced the number of PCIs among the three countries. In the United Kingdom, research funding bodies, such as the Medical Research Council (MRC) and the NIHR, require all applicants to state how they will involve patients and members of the public in the design of the research study, which suggests the culture for patient involvement in medical research is also reasonably well established in the United Kingdom. Nevertheless, this has not yet translated into the establishment of PCIs. Indeed, we found only one funded research project that was using a digital platform.

Furthermore, the differences in the way that health care is organized may be an additional explanatory factor, but this would need further exploration. The US system is rather unique in the way it organizes health care and this may be tied into the more populous PCI landscape in the country. We are aware that patients in the United States are often engaged around social entrepreneurship in health care and play key roles in initiating new research projects [59]. There may also be different attitudes between the three countries toward the utilization of ICT and direct-to-consumer services. Another consideration is the population size. Although the explanatory power is probably somewhat weak, the larger number of PCIs in the United States may, in part, reflect the much larger population size compared to Japan and the United Kingdom.

Future research is needed to disentangle the factors impacting the establishment and sustainability of PCIs in order to better understand the underlying reasons for the differences between these countries.

### Limitations and Future Challenges

In this paper, we investigated PCIs, which enable the active involvement of patients, by conducting a scoping review to understand this new field that has not yet been conceptually established. Therefore, there are a number of limitations to this work.

First, due to limitations in the search method, some PCIs might have been overlooked in our search. For example, any PCI-related documents that do not contain our keywords indicating active involvement of patients in the titles or abstracts would have been extremely difficult to detect. This would also hold for the website search results. We suggested that consistent definition and terminology of participant involvement should be established to overcome these methodological limitations. Second, any PCIs that do not publish their research in scientific articles, or those whose websites do not appear in any search engine results, would not have been found. Further, research articles and websites that were published after our search period would also have not been included in our findings.

For future research, PCIs implemented in countries other than those focused on in this study also deserve attention. For instance, other English-speaking countries, such as Canada, Australia, and New Zealand, where there is a push toward patient and public involvement in medical research could be prioritized in future research. Furthermore, it may be desirable to seek a method to evaluate PCIs by a variety of means, such as careful observation, surveys of participant opinions, and exploration of theoretical considerations. Such results will offer insights to further improve ongoing PCIs and aid the establishment of new ones in the future.

We can also propose possible ideas for moving forward the area of PCIs. In this review, we have documented the important activities of PCIs; at the same time, we have noted that there are still only a small number of projects that meet the criteria we have set for patient involvement. Therefore, the future challenge in the field is how to expand the number of PCIs and also the level of patient involvement that these platforms enable. Ideas for improvement can include the following: (1) wider dissemination of information about the value of PCIs for patients and patient organizations, (2) encouragement of networking between PCIs to facilitate the adoption of good practices, and (3) increase of support by government bodies for PCIs.

### Conclusions

A scoping review was sufficient to capture recent trends in PCIs designed to facilitate medical research. We identified 21 PCIs currently operating in the United States, the United Kingdom, and Japan. This review contributes to a better understanding of the concept of *active involvement* by patients that can be facilitated by PCIs, by clarifying the various modes of communication and decision making. Although it is an emerging initiative in medical research, PCIs have the potential to facilitate fruitful collaboration between participants and researchers.

## Appendix

Multimedia Appendix 1 The search history of the literature database search.

Multimedia Appendix 2 The list of data elements extracted from relevant articles and information on websites of each participant-centric initiative (PCI).

Multimedia Appendix 3 Detailed characteristics of identified participant-centric initiatives (PCIs).

Multimedia Appendix 4 The list of participant-centric initiatives (PCIs) that had not come out of our original literature search.

## Abbreviations

CINAHL: Cumulative Index of Nursing and Allied Health Literature
Embase: Excerpta Medica Database
HAEi: Hereditary Angioedema International
ICT: internet and communication technology
IPBS: Interdisciplinary Program for Biomedical Sciences
JSPS: Japan Society for the Promotion of Science
KAKENHI: Grants-in-Aid for Scientific Research
MEDLINE: Medical Literature Analysis and Retrieval System Online
MeSH: Medical Subject Headings
MRC: Medical Research Council
NHS: National Health Service
NIH: National Institutes of Health
NIHR: National Institute for Health Research
PCI: participant-centric initiative
PCORI: Patient-Centered Outcomes Research Institute
PEER: Promise for Engaging Everyone Responsibly
PRISMA: Preferred Reporting Items for Systematic Reviews and Meta-Analyses
RUDY: Rare and Undiagnosed Diseases Study
US HAEA: US Hereditary Angioedema Association
V-PPRN: Vasculitis Patient-Powered Research Network
