# Supplementary material for: Landscape of Participant-Centric Initiatives for Medical Research in the United States, the United Kingdom, and Japan: Scoping Review
Source: J Med Internet Res. 2020 Aug 4;22(8):e16441. doi: 10.2196/16441 (PMC7435629; doi:10.2196/16441)
Supplement: Multimedia Appendix 1 [file jmir_v22i8e16441_app1.docx]

| **#** | **Searches** | **Results** |
| --- | --- | --- |
| 1 | Community-Based Participatory Research/ | 3352 |
| 2 | patient participation/ | 22379 |
| 3 | 1 or 2 | 25657 |
| 4 | (participant* adj (centric or centered or centred or engage* or involve* or collaborat* or partner* or led or driven or initiat* or oriented)).ti,ab. | 2035 |
| 5 | exp computer systems/ | 161399 |
| 6 | exp database, factual/ | 106160 |
| 7 | online systems/ | 8023 |
| 8 | Registries/ | 71555 |
| 9 | 5 or 6 or 7 or 8 | 329528 |
| 10 | exp Biomedical research/ | 244084 |
| 11 | exp health services research/ | 151387 |
| 12 | 10 or 11 | 346778 |
| 13 | 3 and 9 | 1244 |
| 14 | 3 or 9 or 12 | 679911 |
| 15 | 4 and 14 | 223 |
| 16 | 13 or 15 | 1461 |
| 17 | remove duplicates from 16 | 1356 |

Multimedia Appendix 1: The search history of the literature database search

1. MEDLINE (11 July 2017)

2. Embase (18 July 2018)

| **No.** | **Query** | **Results** |
| --- | --- | --- |
| #1 | 'participatory research'/de | 3662 |
| #2 | 'patient participation'/de | 21873 |
| #3 | #1 OR #2 | 25435 |
| #4 | participant* NEXT/1(centric OR centered OR centred OR engage* OR involve* OR collaborat* OR partner* OR led OR driven OR initiat* OR oriented) | 2366 |
| #5 | 'computer system'/de | 24108 |
| #6 | 'mobile phone'/exp | 15490 |
| #7 | 'software'/de | 199103 |
| #8 | 'web browser'/de | 4980 |
| #9 | 'factual database'/exp | 51659 |
| #10 | 'online system'/de | 21554 |
| #11 | 'disease registry'/de | 9915 |
| #12 | 'registration'/de | 23043 |
| #13 | 'register'/de | 98100 |
| #14 | 'cancer registry'/de | 25731 |
| #15 | #5 OR #6 OR #7 OR #8 OR #9 OR #10 OR #11 OR #12 OR #13 OR #14 | 442773 |
| #16 | 'research'/exp | 747484 |
| #17 | 'health services research'/de | 30461 |
| #19 | #16 OR #17 | 774546 |
| #20 | #3 AND #15 | 771 |
| #21 | #3 OR #15 OR #19 | 1207499 |
| #22 | #4 AND #21 | 415 |
| #23 | #20 OR #22 | 1179 |
| #24 | #23 AND ('conference abstract'/it OR 'editorial'/it OR 'letter'/it) | 237 |
| #25 | #23 NOT #24 | 942 |

3. CINAHL (11 July 2017)

| **Search ID#** | **Search Terms** | **Search Options** | **Results** |
| --- | --- | --- | --- |
| S1 | (participant* N1 (centric or centered or centred or engage* or involve* or collaborat* or partner* or led or driven or initiat* or oriented)) |  | 2,824 |
| S2 | (MH "Consumer Participation") |  | 14,391 |
| S3 | (MH "Internet+") |  | 112,758 |
| S4 | (MH "Communications Media+") |  | 429,300 |
| S5 | (MH "Registries, Disease") OR (MH "Registries, Implant") OR (MH "Registries, Organ") OR (MH "Registries, Trauma") |  | 8,209 |
| S6 | (MH "Researcher-Subject Relations") |  | 972 |
| S7 | (MH "Health Services Research") |  | 11,423 |
| S8 | (MH "Clinical Research+") |  | 10,977 |
| S9 | S2 OR S3 OR S4 OR S5 OR S6 OR S7 OR S8 |  | 494,495 |
| S10 | S1 AND S9 |  | 608 |
| S11 | S10 | **Limiters** - Research Article | 463 |
| S12 | S10 | **Limiters** - Publication Type: Meta Analysis, Meta Synthesis, Review, Systematic Review | 38 |
| S13 | S11 OR S12 |  | 467 |

4. PsycINFO (11 July 2017)

| **#** | **Searches** | **Results** |
| --- | --- | --- |
| 1 | client participation/ | 1743 |
| 2 | (participant* adj (centric or centered or centred or engage* or involve* or collaborat* or partner* or led or driven or initiat* or oriented)).ti,ab. | 2685 |
| 3 | 1 or 2 | 4418 |
| 4 | exp COMPUTERS/ | 17652 |
| 5 | DATABASES/ | 3406 |
| 6 | exp INTERNET/ | 26981 |
| 7 | exp WEBSITES/ | 4528 |
| 8 | exp mobile devices/ | 4854 |
| 9 | exp Social Media/ | 8935 |
| 10 | or/4-9 | 59773 |
| 11 | 3 and 10 | 175 |

5. Ichushi-Web (18 July 2017)

| **#** | **Searches** | **Results** |
| --- | --- | --- |
| #1 | @登録/TH | 8,890 |
| #2 | レジストリ/TA | 1,099 |
| #3 | registr/TA | 2,624 |
| #4 | #1 or #2 or #3 | 10,285 |
| #5 | 患者/TA | 980,072 |
| #6 | #4 and #5 | 2,574 |
| #7 | 参加/TA or 参画/TA or 主体/TA | 59,688 |
| #8 | #6 and #7 | 96 |
