# Supplementary material for: Landscape of Participant-Centric Initiatives for Medical Research in the United States, the United Kingdom, and Japan: Scoping Review
Source: J Med Internet Res. 2020 Aug 4;22(8):e16441. doi: 10.2196/16441 (PMC7435629; doi:10.2196/16441)
Supplement: Multimedia Appendix 2 [file jmir_v22i8e16441_app2.docx]

Multimedia Appendix 2: the list of data elements which were extracted

1. Name of organization
2. Types of organization
3. Launch year
4. Location
5. International research mandates
6. Number of users
7. Focus of registration
8. Categories of diseases and conditions
9. Device

a. PC

b. Smartphone

c. Wearables

1. Collaborators or funding organization
2. Types of data
3. Purposes of the study
4. Numbers of Publication Uploaded
5. Types of consent

a. Specific consent

b. Broad consent

c. Dynamic consent

1. Procedure of electronic consent
2. Methods of participant involvement
3. Remarkable activities of involvement
4. Use of social media
