# Supplementary material for: Landscape of Participant-Centric Initiatives for Medical Research in the United States, the United Kingdom, and Japan: Scoping Review
Source: J Med Internet Res. 2020 Aug 4;22(8):e16441. doi: 10.2196/16441 (PMC7435629; doi:10.2196/16441)
Supplement: Multimedia Appendix 4 [file jmir_v22i8e16441_app4.docx]

Multimedia Appendix 4: the list of PCIs that had not come out in our original literary search.

| 1. RUDY JAPAN (Japan) | https://rudy.hosp.med.osaka-u.ac.jp/ (Accessed 18 September 2019) |
| --- | --- |
| 1. The Cerebral Palsy Research Network (US) | https://cprn.org/ (Accessed 18 September 2019) |
| 1. Cystic Fibrosis Foundation Patient Registry (US) | https://www.cff.org/Research/Researcher-Resources/Patient-Registry/ (Accessed 18 September 2019) |
| 1. National Alopecia Areata Registry (US) | https://www.naaf.org/research/alopecia-areata-registry (Accessed 18 September 2019) |
| 1. The Preeclampsia Registry (US) | https://www.preeclampsiaregistry.org/ (Accessed 18 September 2019) |
| 1. UK MS registry (UK) | <https://ukmsregister.org/>  (Accessed 13 February 2020) |
